# More than a virus: a qualitative study of the social implications of hepatitis B infection in China

**DOI:** 10.1186/s12939-017-0637-4

**Published:** 2017-08-01

**Authors:** J. Wallace, M. Pitts, C. Liu, V. Lin, B. Hajarizadeh, J. Richmond, S. Locarnini

**Affiliations:** 1 Australian Research Centre in Sex, Health and Society, La Trobe University, 215 Franklin Street, Melbourne, Victoria 3000 Australia; 2 China Health Program, La Trobe University, Melbourne, Australia; 3 Department of Public Health, La Trobe University, Melbourne, Australia; 4 The Kirby Institute, UNSW Australia (University of New South Wales), Sydney, Australia; 5 Victorian Infectious Diseases Reference Laboratory, Director, WHO Regional Reference Laboratory for Hepatitis B, Doherty Institute, Melbourne, Australia

**Keywords:** China, Hepatitis B, Social impact, Stigma, Health care access, Qualitative study

## Abstract

**Background:**

China has the largest absolute number of people living with hepatitis B with up to 300,000 people estimated to die each year from hepatitis B related diseases. Despite advances in immunisation, clinical management, and health policy, there is still a lack of accessible and affordable health care for people with hepatitis B. Through in-depth interviews, this study identifies the personal, social and economic impact of living with hepatitis B and considers the role of stigma and discrimination as barriers to effective clinical management of the disease.

**Methods:**

Semi-structured qualitative interviews were held with 41 people living with hepatitis B in five Chinese cities. Participants were recruited through clinical and non-government organisations providing services to people with hepatitis B, with most (*n* = 32) being under the age of 35 years.

**Results:**

People living with hepatitis B experience the disease as a transformative intergenerational chronic infection with multiple personal and social impacts. These include education and employment choices, economic opportunities, and the development of intimate relationships. While regulations reducing access to employment and education for people with hepatitis B have been repealed, stigma and discrimination continue to marginalise people with hepatitis B.

**Conclusions:**

Effective public policy to reduce morbidity and mortality associated with hepatitis B needs to address the lived impact of hepatitis B on families, employment and educational choices, finances, and social marginalisation.

## Background

An estimated 93 million people live with chronic hepatitis B in China, with 300,000 people dying each year as a result of hepatitis B-related complications [1–3]. In spite of the effective implementation of the vaccination program [3–5], China experiences the highest liver cancer incidence in the world as a result of hepatitis B, which accounts for around 55% of global liver cancer deaths [6, 7].

The vast majority of people with chronic hepatitis B in China were infected at birth or in infancy [2]. The Chinese government incorporated hepatitis B vaccination into the national planned immunization scheme for children in 2002 [3], with a subsequent decrease in prevalence among children younger than 1 year from 9.02% in 1992 to 0.69% in 2006. The reduction of mother to child transmission has resulted in an increase in the proportion of people infected through sexual transmission or sharing of injecting equipment, within a context of a significantly reduced number of acute infections [3].

Viral hepatitis has been acknowledged as an infection of global consequence. The World Health Assembly has recommended that national governments develop a strategic political response to the infection, and the World Health Organization last year released the Global Health Sector Strategy on Viral Hepatitis [8]. This strategy provides a target of eliminating viral hepatitis as a public health threat by 2030. Given the size of the epidemic, the development of effective public policy in China that responds to the needs of people with hepatitis B will be pivotal in reaching this target.

The Chinese health system is funded through separate rural and urban health insurance systems, resulting from the 2009 health reforms. Over the past decade, China has achieved almost universal coverage of government subsidized social health insurance programs, with the share of out-of-pocket payments in total health expenditure dropping from around 50% to about 30%. However, there is no clear evidence showing a significant decline of the economic burden of patients, if any, due to rapidly inflated medical expenses [9] and out-of-pocket expenses remain a serious concern for access to treatment services.

Clinical management reduces the morbidity and mortality associated with the infection [10, 11]. Clinical management guidelines have been developed by the Chinese Society of Infectious Diseases and the Chinese Society for Hepatology [12]. Despite improvements in pharmaceutical treatments there is an increasing number of hepatitis B–related deaths [13, 14] and only a small proportion of people with chronic hepatitis B access and receive treatment in China [15]. The full range of pharmaceutical treatments listed by the American Association for the Study of the Liver are available in China, although access to the first-line drugs such as entecavir and interferon is limited by the high cost to the patient [16]. While treatment is available through hospital-based specialist services, only 19% of people with hepatitis B have ever accessed treatment, with barriers including limited reimbursement and other costs related to testing [1, 15, 17, 18]. There is poor adherence to monitoring, with one report of only 33% of 3257 patents with hepatitis B followed up after referral [19]. People with hepatitis B in China experience a lower quality of life than other countries given the impact of stigma and discrimination on employment, marriage and education [20].

Hepatitis B has been described as an economically catastrophic illness in China, with the direct costs of hepatitis B and its related diseases exceeding 40% of household annual income increasing in more advanced liver diseases [21]. The costs are particularly significant for people with hepatitis from rural areas where it is estimated that less than 5% of patients can afford treatment for 1 year compared with approximately 40% of patients in more developed areas [15]. A survey of disease burden in Shanghai showed that the annual direct and indirect medical costs for each patient with chronic hepatitis B were US$3000 with a per capita Gross Domestic Product at that time of around US$1000 per annum [17].

Hepatitis B has social implications for people living with the virus in China particularly through social marginalisation resulting from discrimination and stigma [1, 22, 23]. Regulations limiting the access of people with viral hepatitis to education and employment were passed in the late 1980s [24] and later repealed, when the China Ministries of Health and of Personnel announced that people with hepatitis B must not be discriminated against [25–28]. While compulsory workplace and school-based health checks that included testing for hepatitis B were cancelled in February 2010 [27, 29], testing for liver function is permitted.

This study aimed to further identify the social impact of chronic hepatitis B for people living with the infection in China. Through semi-structured interviews, we sought to understand the personal, interpersonal, economic and social consequences of the disease in the context of a rapidly changing environment. This type of investigation can provide evidence of systemic gaps and barriers that are often not apparent from clinical or epidemiological research. The experiences and needs identified by these findings can form the basis for developing effective policy and programme interventions.

## Methods

A total of 41 semi-structured qualitative interviews were conducted by the lead author with people with hepatitis B living in China. Semi-structured interviews were chosen to provide flexibility to respond to issues raised by the participants. The interview schedule included the following prompts:

- How did you find out you had hepatitis B?
- Who told you that you had viral hepatitis, and how were you told?
- What information were you given when you were told you had hepatitis B?
- Are you being treated for hepatitis B? Is your infection being monitored by a doctor or specialist?
- Has finding out that you have hepatitis B changed your life? How?
- What information would have been useful for you when you found out you had hepatitis B?
- What other information do you need to help you deal with having hepatitis B?

Interviews were conducted with 41 people, of whom 12 were women. Face to face interviews took place in Guangzhou (*n* = 14), Dong Guan (*n* = 2), Shanghai (*n* = 11), and Beijing (*n* = 13) and one telephone interview conducted in Chongqing. Twelve participants were recruited through hospital based clinical services in Beijing and Guangzhou, with the remainder (*n* = 29) through non-government organisations. The majority of participants were under the age of 35 years, see Table 1.

**Table 1:** Age of participants

Age range	No. of people
20–25 years	8
25–30 years	14
30–35 years	10
35–40 years	4
40–45 years	1
45–50 years	2
50–55 years	1
55–60 years	1

Interviews were electronically recorded and conducted in Mandarin through an interpreter: questions were asked in English, interpreted and responded to in Mandarin, and the response then interpreted into English. Three interviews were conducted in English at the request of the participant. Additional clarifying explanations were provided by the interpreter in consultation with the participant. The length of the interviews ranged from 25 to 90 min and interviews were transcribed by the lead investigator. To validate the interpretation, all Mandarin responses were translated using an independent translation service, with no additional findings uncovered by this process.

The analysis of the data followed the stages outlined by Braun and Clarke [30], with data coding and analysis conducted by the first author using a thematic analysis [31]. The analysis began during the interviews and progressed through the careful reading of transcripts. Transcripts were initially and exhaustively coded by hand, and then entered into NVivo 10, (QSR International Pty Ltd., VIC, Australia). Themes were identified and further refined through discussion with the team. Ethical approval for the study was obtained from the La Trobe University Human Research Ethics Committee (14–006). Participation in the research was voluntary and all participants provided informed consent to their participation in the study. The interpreter signed a confidentiality agreement.

## Results

Analyses of the interviews yielded inter-connected themes. These were that hepatitis B is understood as an intergenerational family condition; that there are a substantial economic and psychological impacts as a consequence of a diagnosis of hepatitis B; disclosure was found to significantly influence employment choices; and, social marginalisation was sometimes a major issue for participants and their families.

### A family disease

Hepatitis B in China occurs most commonly through mother to child transmission. The social and familial implications of this can be devastating and long lasting, affecting multiple generations within the family:

*My parents have it, my relatives on my mother’s side have it, two of my mother’s siblings. My mother’s younger sister and younger brother and younger brother’s daughter have it. My father died of liver cancer when I was around 16 years old (Male, 25-30yo, Guangzhou).*

*My mother transmitted that disease to my father when they got married … my uncle also has it … besides that my grandmother has it … and my grandmother’s elder sisters (Female, 20-25yo, Shanghai).*

However, the frequency with which multiple family members can be affected can also, paradoxically, lead to an alternative perspective. Some participants reported that because of the familial nature of the infection, hepatitis B was viewed as unexceptional and that this could reduce anxiety:

*I thought it was just one of the common diseases … in my family there were a few relatives who had this type of disease, so they think it can be treated … my mother has it, my father was a carrier, but was cured before I was born (Male, 20-25yo, Guangzhou).*

This commonality and subsequent acceptance was not experienced by all participants, with one man noting that:

*My relatives were afraid of getting infected and asked me not to go to their home again (Male, 35-40yo, Guangzhou).*

### Economic impact

Sometimes, the diagnosis of hepatitis B in an individual triggered a process where other family members were tested for the virus and subsequently diagnosed. Several participants noted this had a devastating economic impact, particularly since treatment is only available through specialist services located in major cities, and where there is limited reimbursement of costs associated with treatment available.

For example, for one participant, his diagnosis led to the diagnosis of other members of his family. This required the family to relocate to a larger city to access treatment services.

*It placed a huge financial pressure on me as my whole family had the same disease, the pressure was unbearable … my wife has to take medication, I have to take medication, my daughter has to take medication (Male, 35-40yo, Guangzhou)*

This experience highlights one of several inequalities experienced by people with hepatitis B who live in rural settings, where there are very few clinical management services available.

China has experienced significant economic structural changes over the past 40 years, with an increasing middle class, industrialisation and the movement of people from rural to urban centres. Several participants reported strong emotional and psychological responses to being diagnosed, within a context of significant family sacrifice for them to improve or consolidate their economic status.

*I came from a rural area, and I was the only one in my family to have the chance to go to university. My mother was a single mother who brought me up. The reason I went to university was because I wanted to find a decent job. I had no idea how I managed to walk out of the hospital. It’s like I was hit by lightning. I didn’t dare to tell my mother (Female, 30-35yo, Guangzhou).*

The intersection between hepatitis B, parental expectation and economic impact affected several participants, particularly in the context of the one-child policy. Other factors included increasing urbanisation and industrialisation and a recognition of the importance of education for the future, not only of the individual, but for the whole family:

*My health condition placed me under a lot of pressure … I couldn’t cope with a heavy workload … I had to ask my family to support me financially. I felt I betrayed their expectations (Male, 25-30yo, Beijing).*

*My parents were peasants; their only hope in their lifetime was for me and brother to go to university (Male, 25-30yo, Shanghai).*

While hepatitis B infection cannot be cured, clinical management including regular monitoring and pharmaceutical treatment can reduce the potential physical impact of the infection. Treatment, when required, is usually lifelong. The economic burden arising from inadequate reimbursement for treatment in China affected the choice of pharmaceutical drugs used by participants, with an economic rather than clinical outcome guiding the choice:

*I need to think about my financial situation when I make decisions. I need to compare the price … I need to find out whether expensive medication is more effective than cheaper ones (Male, 30-35yo, Beijing).*

The use of sub-optimal medicines such as Lamivudine, was recommended to one participant because of its cost:

*I found a senior professor … after seeing my results, he recommended … anti-viral treatments. He asked me about my family’s financial situation and what type of treatment I could afford. I told him my family’s financial situation’s not very good, so he recommended me to have Lamivudine (Male, 35-40yo, Guangzhou).*

As one man noted, if he was wealthy, he *could have the best treatment in the world (Male, 20-25yo, Guangzhou).*

### Employment choices

The work-related impact of hepatitis B infection was significant, particularly as most participants were under the age of 35 years, for whom employment and the development of a career were key issues. While legislation in China that limited access to education and employment as a result of hepatitis B infection has been repealed, the impact of testing and a diagnosis of hepatitis B remain substantial.

Several participants noted that continued workplace testing for hepatitis B limited their choice of employment and the scope of the employers available to them:

*I was forced to work in the small (factory), because large factories conduct health checks … To enter big factories, I might need to bribe the staff by giving them a couple thousand RMB (Male, 35-40yo, Guangzhou).*

While participants reported relocating to major cities to access clinical services, employment was also the rationale for relocation.

*I can only find jobs with which I am not totally satisfied. The reason I came to Beijing to seek jobs was because I thought Beijing’s policy was more flexible (Male, 25-30yo, Beijing).*

### Marginalisation

Disclosing that a person has hepatitis B risks marginalisation with participants describing the complex process of negotiation that occurred:

*I only tell my close friends… there are some who still feel uncomfortable with it, but just a couple of them, not many. But the others, when they mention hepatitis in casual conversation, they seem to be very scared about it (Female, 20-25yo, Guangzhou).*

The motivation for some participants to disclose their infection resulted from their concern of transmitting hepatitis B to others, even when these fears had little scientific credibility, public health veracity or cultural credence.

*I told people who are close to me and whom I used to share food with, as I don’t want to get them infected…. When we have meals together, they will serve me food to prevent me from touching the food (Female, 20-25yo, Guangzhou).*

Over half of the participants (*n* = 32) with hepatitis B interviewed for this study were under the age of 35 years and they discussed the challenges of having hepatitis B and developing intimate relationships.

*Recently I have been looking to start a relationship. I have met four or five boys but because of this disease, I was rejected by them all (Female, 25-30yo, Shanghai).*

These concerns also resulted in familial issues through Chinese cultural norms where relationships and marriages are negotiated between families.

*My girlfriend’s family didn’t want us to be together any more after they found out about my condition. They told my girlfriend to break up with me. But my girlfriend insisted on staying with me (Male, 25-30yo, Beijing).*

Significant emotional and psychological responses were described by participants who noted how being diagnosed with hepatitis B had changed their lives through feeling marginalised.

*You’re different. You’re a carrier … I was silent (Male, 30-35yo, Guangzhou).*

*I hide myself (Female, 25-30yo, Guangzhou).*

*I carry a burden (Male, 30-35yo, Shanghai).*

A fear and lack of information about hepatitis B was common, both among people with the infection, and more broadly within the community as a whole.

*I was very depressed, I was very concerned about this situation, I totally believed that I could get everybody [infected] including the people who had a close relationship with me (Male, 30-35yo, Guangzhou).*

## Discussion

This study sought to investigate the breadth of the personal impact of hepatitis B infection, and to identify the social issues for people as they respond to the infection. These findings can be used to contribute and improve policies and programs seeking to reduce the burden of infection on people infected with hepatitis B in China.

For people in China, living with hepatitis B has extensive social as well as clinical implications. Stigma and discrimination have been identified as key social issues for people with hepatitis B in China [32–38], and who have migrated from China to other countries [35–38]. While recognising that stigma and discrimination needs to be acknowledged as an essential element in the lives of people with hepatitis B, this study sought to systematically identify how these and other social issues affected access to health services. This extends earlier survey based studies by focussing on the lived experiences as described by our participants [27, 39].

Hepatitis B was regarded as an intergenerational chronic disease. For many participants, the familial impact was a strong influence on how they responded to diagnosis and management of the virus. This relationship between their personal infection, and the experience of the infection within their family needs further investigation, particularly the impact of these experiences on accessing clinical services for the infection.

One consequence of the intergenerational and familial impact of the infection is that diagnosis of one person in the family led to other family members being diagnosed, requiring clinical intervention as a result of this diagnosis. Even when family members were not infected, a diagnosis often led to a substantial economic and social impact, particularly for people from rural areas seeking to strengthen their economic standing or where people had to relocate to access clinical services. These implications have not been noted previously, and have important implication for the management of the disease.

While hepatitis B affects individuals physically [10] and psychologically [40, 41], our findings extend understanding of the effects on education and employment choices. These flow on to individual and familial economic impact, career development, relationships, and residential location. These responses occur within the dynamic context of contemporary China with rapid social, cultural and economic changes.

Previous evidence of the economic impact of hepatitis B noted its significant impact [15, 21]. This study adds to the implications of this impact of individuals with hepatitis B. The economic impact of the infection are a result of policy choices by government and clinical specialists. Inadequate treatment reimbursement means that clinical choices are, for some patients, dominated by economic need.

While discriminatory regulations have been repealed, people with hepatitis B still report that their infection affects employment and educational choices, relationships and their role within society. There are a range of issues influencing the social status of people with hepatitis B including a previously recognised poor understanding of the infection among the general public and health care workers [42, 43], the poorly regulated nature of government and non-government funded clinical services, and the unsystematic testing and diagnosis of hepatitis B. The lack of accurate information within the general community affects the willingness of people with viral hepatitis to disclose their infection to friends, colleagues, and for some, their families. This essentially marginalises people with hepatitis B from participation in the wider society, and limits a person’s willingness to participate in the clinical management of the infection.

Recommendations from this study include the development of a national strategic response to hepatitis B that specifically acknowledges and addresses the social implications of the infection. The strategic response needs to recognise and respond to the impact of hepatitis B occurring within families, and the marginalisation and limited education and employment opportunities. Actions from the plan would include the provision of authoritative and consistent information campaigns targeting the general public, health care workers and people with hepatitis B, and the identification of regulations that inhibit the full involvement of people with hepatitis B within the community.

There are several limitations to this study including the representativeness of the sample, the limited number of recruitment locations, a focus on urban centres, and the use of an interpreter. Nonetheless this study generated novel and deeper understandings of the lived experience of people with hepatitis B in contemporary China. It extends the earlier work which had a clinical focus and a clinic based recruitment strategy [39].

## Conclusions

Policy and programmes designed to reduce the burden of chronic viral hepatitis in China need to recognise that hepatitis B has significant social implications. A comprehensive national response to the infection needs to address the social as much as the clinical management and public health or preventive aspects of hepatitis B.
